# Establishment and validation of a prognostic nomogram for long-term low vision after diabetic vitrectomy

**DOI:** 10.3389/fendo.2023.1196335

**Published:** 2023-08-25

**Authors:** Haoxin Guo, Zhaoxiong Wang, Zetong Nie, Xiang Zhang, Kuan Wang, Naxin Duan, Siqiong Bai, Wenbo Li, Xiaorong Li, Bojie Hu

**Affiliations:** ^1^ Tianjin Key Laboratory of Retinal Functions and Diseases, Tianjin Branch of National Clinical Research Center for Ocular Disease, Eye Institute and School of Optometry, Tianjin Medical University Eye Hospital, Tianjin, China; ^2^ Department of Ophthalmology, Tianjin Baodi Hospital, Tianjin, China; ^3^ Baodi Clinical College, Tianjin Medical University, Tianjin, China; ^4^ Department of Retinal Disease, Cangzhou Eye Hospital, Cangzhou, China

**Keywords:** proliferative diabetic retinopathy, vitrectomy, nomogram, risk prediction model, low vision

## Abstract

**Purpose:**

We aimed to evaluate the risk factors and develop a prognostic nomogram of long-term low vision after diabetic vitrectomy.

**Methods:**

This retrospective study included 186 patients (250 eyes) that underwent primary vitrectomy for proliferative diabetic retinopathy with a minimum follow-up period of one year. Patients were assigned to the training cohort (200 eyes) or validation cohort (50 eyes) at a 4:1 ratio randomly. Based on a cutoff value of 0.3 in best-corrected visual acuity (BCVA) measurement, the training cohort was separated into groups with or without low vision. Univariate and multivariate logistic regression analyses were performed on preoperative systemic and ocular characteristics to develop a risk prediction model and nomogram. The calibration curve and the area under the receiver operating characteristic curves (AUC) were used to evaluate the calibration and discrimination of the model. The nomogram was internally validated using the bootstrapping method, and it was further verified in an external cohort.

**Results:**

Four independent risk factors were selected by stepwise forward regression, including tractional retinal detachment (β=1.443, OR=4.235, P<0.001), symptom duration ≥6 months (β=0.954, OR=2.595, P=0.004), preoperative BCVA measurement (β=0.540, OR=1.716, P=0.033), and hypertension (β=0.645, OR=1.905, P=0.044). AUC values of 0.764 (95% CI: 0.699-0.829) in the training cohort and 0.755 (95% CI: 0.619-0.891) in the validation cohort indicated the good predictive ability of the model.

**Conclusion:**

The prognostic nomogram established in this study is useful for predicting long-term low vision after diabetic vitrectomy.

## 1 Introduction

Diabetic retinopathy (DR) has become the leading cause of blindness among working-age adults (20–79 years old) (1). In 2020, the worldwide prevalence of DR was approximately 103.12 million, and it is anticipated that by 2030, this number would rise to 129.84 million, and by 2045, it will reach 160 million (2), illustrating the growing burden of DR as a public health issue. Proliferative DR (PDR), characterized by neovascularization, can result in considerable vision loss owing to severe complications (3).

The development of microincision vitrectomy surgery has greatly improved the precision, safety, and efficiency of intraocular surgery in recent years. Pars plana vitrectomy (PPV) has been widely used in the treatment of PDR complications, including un-clearing vitreous hemorrhage (VH) (1–6 months) and tractional retinal detachment (TRD) involving or threatening the macula (4). This surgery is performed to remove VH, peel off the fibrovascular membrane, and reattach the retina. The life expectancy of people with diabetes is increasing, and their demands for a higher quality of life are rising (5). Therefore, the purpose of PPV for PDR is not only intended to prevent blindness, but also to sustain good visual function long-term.

Before surgery, a better understanding of prognostic factors related to long-term vision after vitrectomy can help clinicians make better surgical decisions, assist patients with PDR in adjusting their psychological expectations, and foster better patient-doctor communication. Previous studies have explored several factors related to postoperative vision; however, the independent variables differ among them (6–9). In this study, we developed and validated a preoperative data-based prognostic nomogram for long-term low vision after diabetic vitrectomy using appropriate sample size and comprehensive medical records. Furthermore, we analyzed the clinical features of PDR patients complicated with chronic kidney disease (CKD).

## 2 Methods

### 2.1 Study design and participants

This is a single-center and retrospective study (registered at ClinicalTrials.gov: NCT05631054). It was approved by the Ethics Committee of Tianjin Medical University Eye Hospital (approval number: 2022KY-27) and was conducted in accordance with the Declaration of Helsinki. We included 186 PDR patients (250 eyes) who underwent primary vitrectomy in Tianjin Medical University Eye Hospital from January 2016 to October 2021. We defined 12 months postoperatively as a long-term period because previous studies have indicated that patients could gain stable visual acuity after this period of time (7, 8). The key inclusion criteria were: (1) >18 years of age; (2) diagnosed with type I or II diabetes mellitus; (3) voluntarily signed an informed consent form; (4) presence of un-clearing VH (1–6 months); and (5) TRD involving or threatening the macular confirmed by ophthalmic examinations. The key exclusion criteria were: (1) a follow-up period of<12 months; (2) prior intraocular surgeries in the study eye (except cataract surgery); (3) missing medical records; (4) combined with non-diabetic complications (e.g., retinal vein occlusion, macular hole, keratopathy); (5) surgeries of the study eye within 3 months prior to the last follow-up (e.g., cataract surgery, intravitreal injection, re-vitrectomy, silicone oil extraction). At a 4:1 ratio, patients were assigned to either the training cohort (200 eyes) or the validation cohort (50 eyes) randomly.

### 2.2 Surgical procedures

Standard 23- or 25-gauge PPV was performed under retrobulbar anesthesia. Intravitreal injection of anti-vascular endothelial growth factor (VEGF) drugs was performed 3–5 days prior to surgery in eyes with broad basement adhesion of the fibrovascular proliferative membrane to the retina. The anterior, posterior, and peripheral vitreous body were substantially removed using a high-speed vitrectomy surgical system, and the posterior vitreous cortex was removed with the aid of triamcinolone acetonide. Moreover, neovascular membranes were dissected and/or peeled off. Endo-laser photocoagulation was finished, and the number of laser shots depended on the preoperative laser status and detached area of the retina. Depending on the condition of the retina, tamponade with a balanced salt solution, air, C3F8 gas, or silicone oil was used. Intravitreal injection of anti-VEGF drugs or dexamethasone intravitreal implant was performed, if necessary. Phacoemulsification and intraocular lens implantation were combined for visually significant cataracts. Generally, silicone oil was removed 3–6 months postoperatively when the retina was stable. The surgical procedures were all performed by one surgeon.

### 2.3 Data collection

We collected data by reviewing the patients’ medical records. As systemic factors, we collected the information on sex, age, diabetes type, diabetes duration, hypertension, dialysis, coronary heart disease, cerebral infarction, and blood test (the details are listed in **Table 1**). The CKD epidemiology collaboration equation was used to calculate the estimated glomerular filtration rate (eGFR) (10). Moreover, the following ophthalmological parameters were collected: symptom duration, best-corrected visual acuity (BCVA) measurement, indication for surgery, history of pan-retinal photocoagulation, and history of anti-VEGF intravitreal injection. The primary outcome was the BCVA measurement at the last follow-up, and the secondary outcomes included the anatomical outcome and occurrence of neovascular glaucoma (NVG) and re-vitrectomy.

**Table 1:** Baseline characteristics of the training cohort.

	Non-low vision (n=111)	Low vision (n=89)	χ^2^/Z/t	P
Sex^a^(Male%)	43(36.8)	29(32.6)	0.812	0.368
Age^b^[M(Q1,Q3),years]	47(38,55)	50(41,59)	1.980	0.048^*^
Diabetes type^a^(type2%)	94(84.7)	84(94.4)	4.745	0.029^*^
Diabetes duration^b^[M(Q1,Q3),years]	10(5,16)	10(8,15)	1.220	0.222
HbA1c^b^[M(Q1,Q3),%]	7.9(6.7,8.9)	7.5(6.6,8.2)	-1.647	0.100
Dialysis^a^[n(%)]	3(2.7)	4(4.5)	0.089	0.766
Hypertension^a^[n(%)]	48(43.2)	54(60.7)	6.006	0.014^*^
CHD^a^[n(%)]	16(14.4)	15(16.9)	0.224	0.636
Cerebral infarction^a^[n(%)]	6(5.4)	7(7.9)	0.492	0.483
TC^b^[M(Q1,Q3),mmol/L]	4.90(4.20,5.50)	5.00(4.07,5.71)	0.408	0.683
TG^b^[M(Q1,Q3),mmol/L]	1.66(1.18,2.29)	1.73(1.26,2.50)	0.372	0.710
PT^b^[M(Q1,Q3),s]	11.6(11.0,12.3)	11.5(10.9,12.1)	-0.645	0.519
APTT^b^[M(Q1,Q3),s]	30.4(28.1,33.3)	29.6(27.4,32.4)	-1.573	0.116
FIB^b^[M(Q1,Q3),g/L]	3.15(2.74,3.61)	3.11(2.74,3.95)	0.652	0.514
BUN^b^[M(Q1,Q3),mmol/L]	5.37(4.10,7.00)	5.79(4.60,7.93)	1.350	0.177
eGFR^b^[M(Q1,Q3), mL/min/1.73 m^2^]	91.49(64.57,105.65)	79.96(54.20,99.67)	-2.396	0.017^*^
HGB^c^($\bar{\text{x}}$± s,g/L)	132 ± 17	130 ± 16	0.964	0.336
Symptom duration^b^[M(Q1,Q3),months]	3(1,6)	5(2,12)	2.909	0.004^*^
logMARBCVA^b^[M(Q1,Q3)]	1.30(0.80,1.85)	1.70(1.28,2.30)	2.431	0.015^*^
TRD^a^[n(%)]	24(21.6)	51(57.3)	26.833	<0.001^**^
History of PRP^a^[n(%)]	42(37.8)	34(38.2)	0.003	0.958
History of anti-VEGF^a^[n(%)]	18(16.2)	13(14.6)	0.098	0.755
Follow-up period^b^[M(Q1,Q3),months]	23(13,44)	36(17,48)	1.435	0.151

a: chi-squared test, b: Mann-Whitney test, c: t-test; ^*^P<0.05, ^**^P<0.01.

HbA1c, glycosylated hemoglobin A1c; CHD, coronary heart disease; TC, total cholesterol; TG, triglyceride; PT, prothrombin time.

APTT, activated partial thromboplastin time; FIB, fibrinogen; BUN, blood urea nitrogen; eGFR, estimated glomerular filtration rate.

HGB, hemoglobin; TRD, tractional retinal detachment; PRP, panretinal photocoagulation; VEGF, vascular endothelial growth factor.

### 2.4 Statistical analysis

Low vision was defined as a BCVA<0.3, according to the standard of the WHO 2015 (8). According to BCVA at the last follow-up, the training cohort was divided into low vision and non-low vision groups for comparative analysis. Regarding the change in BCVA, an increase of ≥0.3 logarithmic minimum angle of resolution (logMAR), a change of<0.3 logMAR, and a decrease of ≥0.3 logMAR were defined as “improvement,” “invariant”, and “worsening”, respectively. For statistical analysis, the decimal visual acuity was transformed to logMAR. Values of 1.85, 2.3, 2.6, and 2.9 logMAR were assigned to counting fingers, hand movement, light perception, and no light perception, respectively (11). We selected 60 mL/min/1.73 m^2^ and 30 mL/min/1.73 m^2^ as the cutoff values for eGFR (12). A value >60 mL/min/1.73 m^2^ (normal eGFR), 30–60 mL/min/1.73 m^2^ (medium eGFR), and<30 mL/min/1.73 m^2^ (low eGFR) indicated borderline/normal renal function, early renal insufficiency, and poor renal function, respectively. For continuous variables, statistical results were displayed as mean ± standard deviation (SD) when they were normally distributed and as the median and interquartile range (IQR) when they were non-normally distributed. Percentage and frequency were used for categorical variables. The statistical analysis of continuous variables was conducted using t-test, Mann-Whitney U test, and analysis of variance, while categorical variables were analyzed using chi-squared test or Fisher’s exact test.

The multivariate binary logistic regression analysis considered variables with a P-value<0.1 from the univariate analysis, and stepwise forward regression was used to explore variables with a P-value<0.05 as potential independent predictors. The variance inflation factor was calculated to test for multicollinearity amongst the independent variables. In accordance with the outcomes of the multivariate logistic regression analysis, a nomogram for estimating the probability of long-term low vision after diabetic vitrectomy was built.

The discrimination capacities of the predictive indicators were described using receiver operating characteristic (ROC) curves. Generally, a value >0.7 of the area under the ROC curve (AUC) is considered to indicate good discrimination. The threshold of the equation was determined by the maximum Youden Index (sensitivity + specificity - 1). The calibration was tested and depicted using the Hosmer-Lemeshow test and calibration curves. The nomogram was internally validated using the bootstrapping approach, and it was externally validated in an external cohort. All statistics were analyzed using IBM SPSS Statistics ver. 25.0 (SPSS, Chicago, IL, USA) and R software ver. 4.0.1 (R Project for Statistical Computing, Vienna, Austria). A two-sided P-value of<0.05 was considered statistically significant.

## 3 Results

### 3.1 Patient characteristics and ophthalmic outcomes

A total of 491 patients underwent primary vitrectomy for PDR at Tianjin Medical University Eye Hospital from January 2016 to October 2021. Among them, 186 patients (250 eyes) who met the eligibility criteria were included in this study. The mean follow-up duration was 32 ± 21 months. For all eyes, the mean postoperative BCVA was 1.53 ± 0.66 logMAR, and it increased to 0.8 ± 0.85 logMAR at the last follow-up (P<0.001). The total incidence of long-term low vision was 43.6%. The rate of improvement, invariant, and worsening of BCVA was 68.8%, 15.2%, and 16.0%, respectively. The rate of the final BCVA of ≥0.7, 0.3–0.7, 0.1–0.3, and<0.1 was 22%, 34.4%, 18.4%, and 25.2%, respectively. Final anatomical success was achieved in 235 eyes (94%). During the follow-up period, 40 eyes (16%) underwent re-vitrectomy for recurrent VH in 31 eyes and retinal detachment in 9 eyes, and 18 eyes (7.2%) developed NVG.

### 3.2 Predictive model and nomogram development

In the training cohort of 200 eyes, low vision occurred in 89 eyes (44.5%). **Table 1** provides a list of the characteristics evaluated in this study. The results indicated that age, diabetes type, hypertension, eGFR, symptom duration, preoperative BCVA measurement, and TRD were significantly related to long-term low vision after diabetic vitrectomy (P<0.05).

Considering the practical clinical application, we stratified three continuous variables: age (≤55 years old, >55 years old), symptom duration (<6 months, ≥6 months), and eGFR (<60 mL/min/1.73 m^2^, ≥60 mL/min/1.73 m^2^). According to stepwise forward regression analysis, four independent predictors were included in the risk prediction model: TRD (β=1.443, OR=4.235, P<0.001), symptom duration ≥6 months (β=0.954, OR=2.595, P=0.004), preoperative BCVA measurement (β=0.540, OR=1.716, P=0.033), and hypertension (β=0.645, OR=1.905, P=0.044). The result of the logistic regression analysis is presented in **Table 2**. The equation of the model is:

**Table 2:** Stepwise multivariate logistic regression for long-term low vision after diabetic vitrectomy.

Predictors	β	S.E	Wald statistic	OR(95%CI)	P
Constant	-2.295	0.496	21.387		<0.001^**^
TRD					
No	0			1	
Yes	1.443	0.331	18.981	4.235(2.212,8.108)	<0.001^**^
Symptom duration≥6m					
No	0			1	
Yes	0.954	0.334	8.135	2.595(1.348,4.997)	0.004^*^
logMARBCVA	0.540	0.253	4.556	1.716(1.045,2.818)	0.033^*^
Hypertension					
No	0			1	
Yes	0.645	0.321	4.041	1.905(1.016,3.572)	0.044^*^

TRD, tractional retinal detatchment; m, months; ^*^P<0.05, ^**^P<0.01.


$$\begin{array}{l} logitP=-2.295+1.443\times (TRD=1)+0.954\times (symptom\;duration\geq 6months=1)+0.540\times \\ (logMARBCVA)+0.645\times (hypertension=1),\\ P=e^{logitP}/(1+e^{logitP}) \end{array}$$

where *logitP* is the linear predictive value and *P* is the predictive probability.

The variance inflation factor of each predictor in this model was<10, indicating there was no multicollinearity among the independent variables. The cutoff score that maximized the Youden index was 0.495 (sensitivity, 62.9%; specificity, 78.4%).

**Figure 1:**
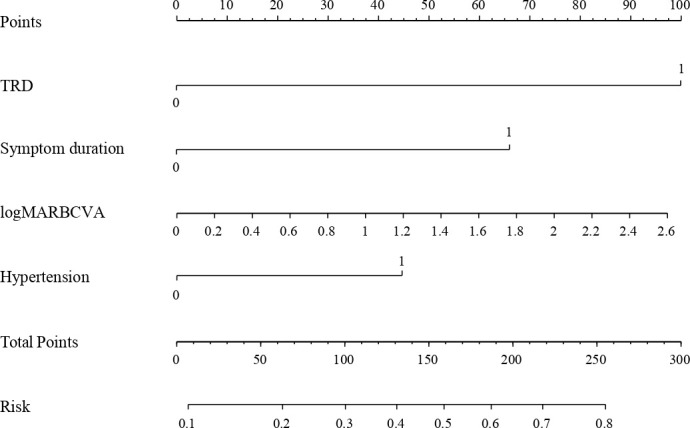
Nomogram for predicting long-term low vision after diabetic vitrectomy.

Then a nomogram was depicted to present the logistic prediction model (**Figure 1**). The total points were calculated according to the conditions of different patients. More total points indicated a higher probability of occurrence of long-term low vision after diabetic vitrectomy.

### 3.3 Validation and evaluation of the nomogram

**Figure 2:**
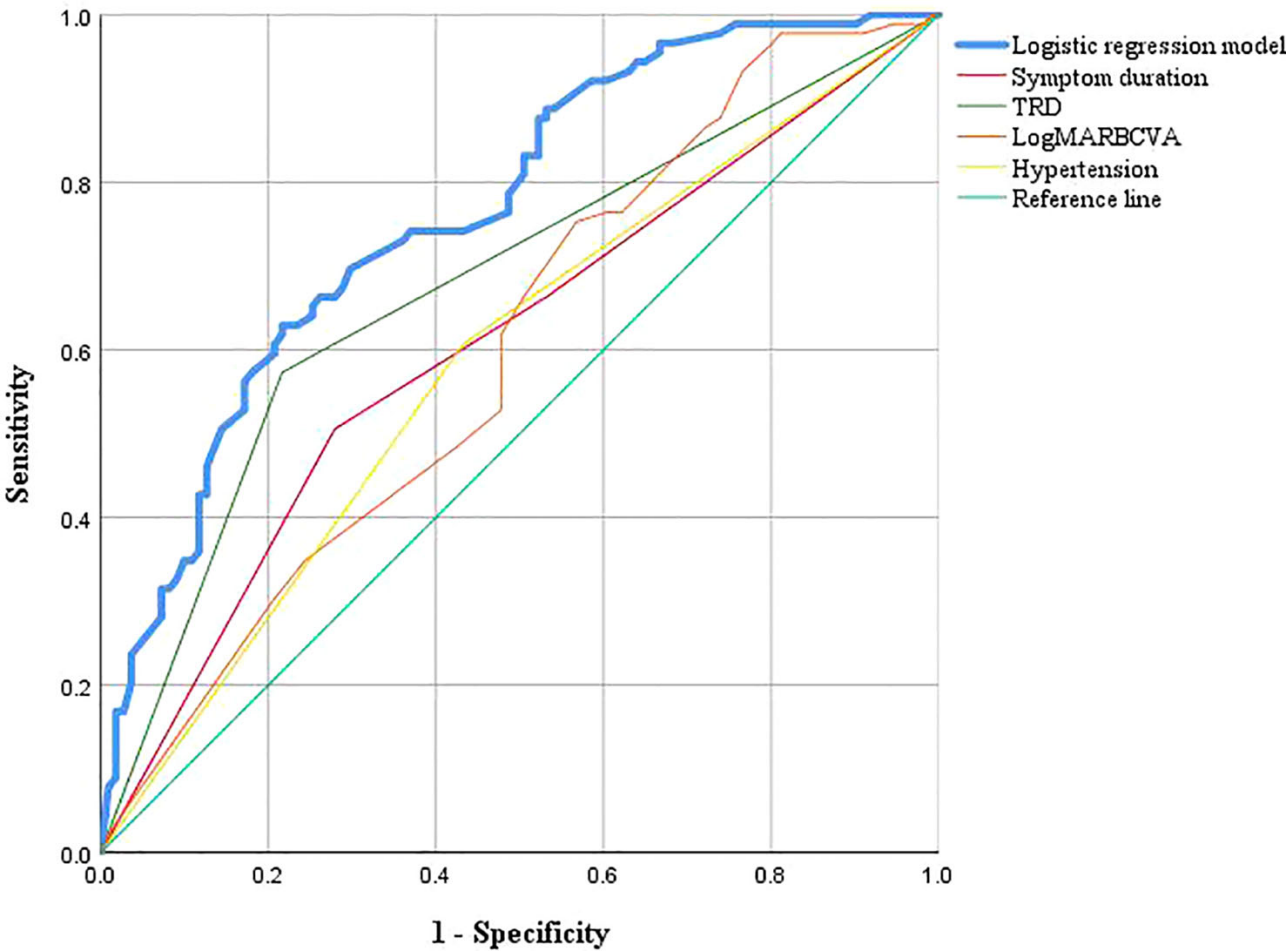
ROC curves of multivate logistic regression model.

**Figure 3:**
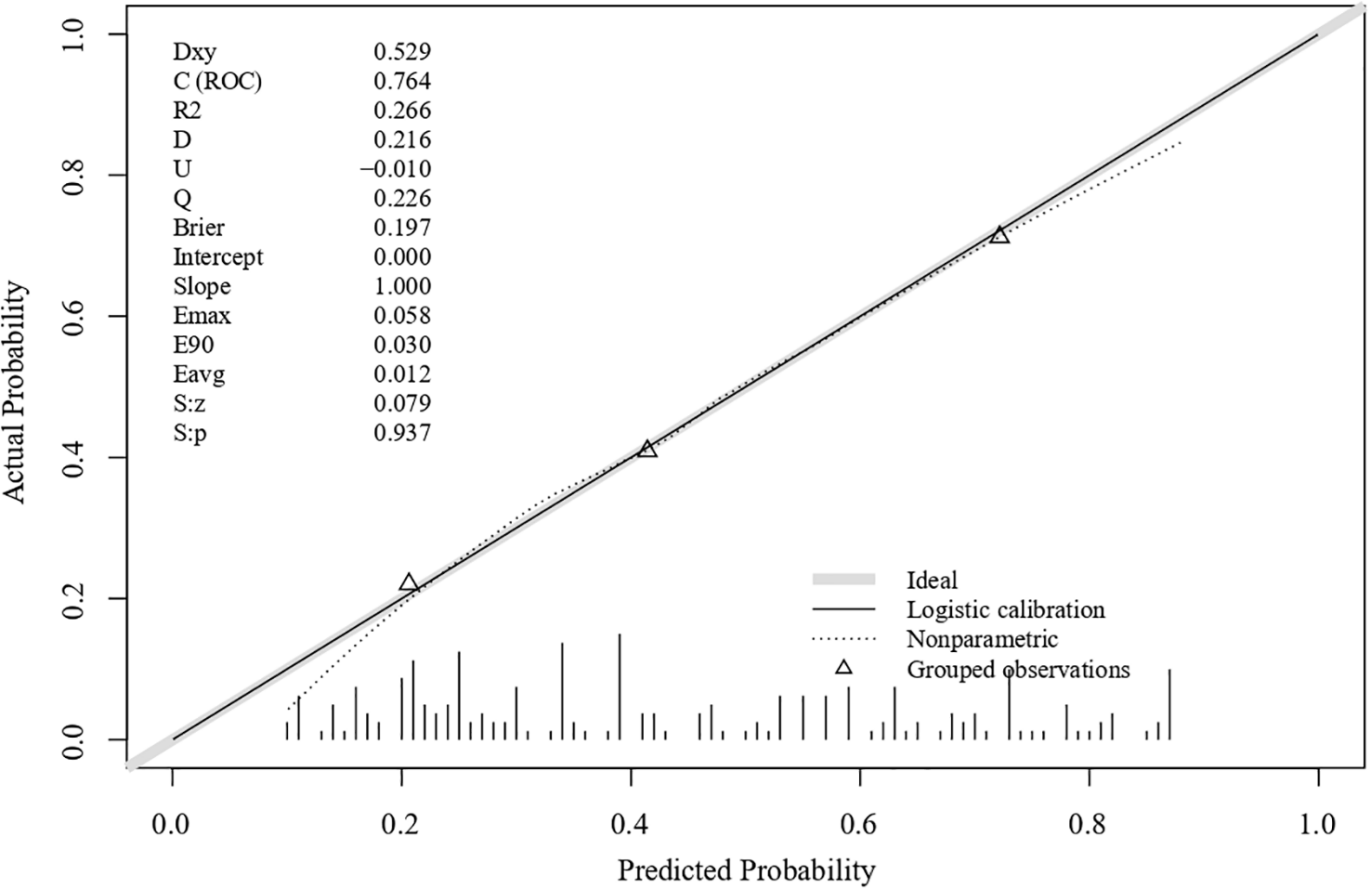
Calibration curve of the risk prediction model.

The ROC curves for the model and each predictor are depicted in **Figure 2**. The discrimination was good, as indicated by the AUC of 0.764 (95% CI: 0.699–0.829). The nomogram also exhibited good calibration on its calibration curve (**Figure 3**). Moreover, the Hosmer–Lemeshow test confirmed the calibration (c^2^ = 8.799, P=0.360). The AUC of the internal validation was 0.747 using bootstrapping with 1,000 replicates. The external validation cohort included 50 eyes, and long-term low vision occurred in 20 eyes (40%). There were no significant differences in all baseline characteristics between the two cohorts (**Table 3**). In the external validation cohort, the model exhibited good prediction accuracy according to the AUC of 0.755 (95% CI: 0.619–0.891).

**Table 3:** Comparison of baseline characteristics of training cohort and validation cohort.

	Training cohort (n=200)	Validation cohort (n=50)	χ^2^/Z/t	P
Sex^a^(Male%)	72(36)	15(30)	0.635	0.426
Age^b^[M(Q1,Q3),years]	49(39,58)	49(36,56)	-1.134	0.257
Diabetes type^a^(type2%)	178(89)	41(82)	1.804	0.179
Diabetes duration^b^[M(Q1,Q3),years]	10(7,16)	10(3,15)	-0.892	0.372
HbA1c^b^[M(Q1,Q3),%]	7.7(6.7,8.6)	7.9(6.7,8.7)	0.352	0.725
Dialysis^a^[n(%)]	7(3.5)	3(6.4)	0.241	0.623
Hypertension^a^[n(%)]	102(51)	24(48)	0.144	0.704
CHD^a^[n(%)]	31(15.5)	10(20)	0.591	0.442
Cerebral infarction^a^[n(%)]	13(6.5)	5(10)	0.733	0.392
TC^b^[M(Q1,Q3),mmol/L]	4.92(4.19,5.50)	5.40(3.99,5.83)	0.870	0.384
TG^b^[M(Q1,Q3),mmol/L]	1.70(1.24,2.35)	1.66(0.97,3.05)	0.324	0.746
PT^b^[M(Q1,Q3),s]	11.5(10.9,12.2)	11.1(10.5,11.7)	-1.944	0.052
APTT^b^[M(Q1,Q3),s]	30.0(27.8,32.6)	29.8(26.8,32.4)	-0.824	0.410
FIB^b^[M(Q1,Q3),g/L]	3.11(2.73,3.79)	3.17(2.68,3.61)	0.053	0.958
BUN^b^[M(Q1,Q3),mmol/L]	5.60(4.38,7.31)	5.85(5.09,8.05)	1.755	0.079
eGFR^b^[M(Q1,Q3), mL/min/1.73 m2]	86.7(57.2,104.1)	87.5(57.2,105.3)	0.102	0.919
HGB^c^($\bar{\text{x}}$ ± s,g/L)	131 ± 16	126 ± 19	1.929	0.055
Symptom duration^b^[M(Q1,Q3),months]	3(1,6)	3(1,6)	-1.084	0.279
logMARBCVA^b^[M(Q1,Q3)]	1.60(1.00,2.23)	1.85(1.15,2.30)	1.144	0.253
TRD^a^[n(%)]	75(37.5)	19(38)	0.004	0.948
History of PRP^a^[n(%)]	76(38)	20(40)	0.068	0.795
History of anti-VEGF^a^[n(%)]	31(15.5)	8(16)	0.008	0.931
Follow-up period^b^[M(Q1,Q3),months]	29(15,45)	19(13,39)	-1.798	0.072

a:chi-squared test, b: Mann-Whitney test, c: t-test; ^*^P<0.05, ^**^P<0.01.

HbA1c, glycosylated hemoglobin A1c; CHD, coronary heart disease; TC, total cholesterol; TG, triglyceride; PT, prothrombin time.

APTT, activated partial thromboplastin time; FIB, fibrinogen; BUN, blood urea nitrogen; eGFR, estimated glomerular filtration rate.

HGB, hemoglobin; TRD, tractional retinal detachment; PRP, panretinal photocoagulation; VEGF, vascular endothelial growth factor.

### 3.4 Renal function-stratified analysis

In the training cohort, eGFR differed significantly only in the univariate analysis between the low and non-low vision groups. We further analyzed the features and prognosis of all the patients with borderline/normal renal function (n=184), early renal insufficiency (n=43), and poor renal function (n=23). For baseline characteristics, patients with worse renal function were shown to have significantly longer symptom duration, more hypertension, lower hemoglobin levels, lower hemoglobin A1c levels, and higher fibrinogen levels (P<0.05). There was no significant difference in preoperative median BCVA measurement (normal eGFR group, 1.7 (1.0–2.0) logMAR; median eGFR group, 1.5 (1.0–2.3) logMAR; low eGFR group, 2.3 (0.8–2.3) logMAR; P=0.178). The long-term BCVA significantly improved in the three groups (P<0.01); however, those with lower eGFR experienced considerably worse vision outcomes (normal eGFR group, 0.4 (0.15–1.0) logMAR; median eGFR group, 0.7 (0.4–1.3) logMAR; low eGFR group, 0.8 (0.4–2.3) logMAR; P=0.011). Furthermore, there was no significant difference in BCVA changes among the groups (P=0.068). After adjusting for symptom duration and hypertension, renal function was still an independent predictor of negative visual outcomes in the multilinear regression analysis (P=0.024). The incidence of NVG in the renal insufficiency group (n=66) was substantially higher than that in the group of patients with borderline/normal renal function (13.6% vs. 4.9%, P=0.018). Finally, incidence of re-vitrectomy in the three groups did not differ significantly from one another (P=0.933).

## 4 Discussion

For serious PDR complications, vitrectomy may be the only approach to improve visual acuity. Microinvasive vitreous surgery and an optimized vitrectomy platform with fluid and pressure control have greatly reduced the complications of PPV; however, some patients cannot fully achieve their ideal visual function long-term after diabetic vitrectomy. The estimated incidences of long-term postoperative BCVA<0.3 and<0.1 are 40% and 20%, respectively (6–8, 13), often failing to meet patient expectations. We have therefore established a prognostic nomogram of long-term low vision after diabetic vitrectomy using preoperative characteristics. Moreover, high-risk patients should be closely monitored to minimize the possibility of long-term vision loss after vitrectomy.

In this study, TRD, symptom duration, preoperative BCVA measurement, and hypertension were identified as independent risk factors for long-term low vision after diabetic vitrectomy. With an AUC value of 0.764, along with 0.747 and 0.755 in the internal and external validation cohorts, the risk prediction model based on the four parameters demonstrated good discrimination. The nomogram provided a graphical representation of mathematical formulas, facilitating understanding and application. The factors in this model are easily obtainable, allowing doctors to make a preliminary evaluation of long-term functional outcomes through routine examinations and have good preoperative communication with patients.

In this model, TRD involving or threatening the macular was regarded as the greatest risk factor. Patients with TRD had a 4.235 times increased incidence of postoperative long-term low vision than individuals without TRD. Although the final retinal reattachment rates have been reported to range from 95–100% (14), the increase in visual acuity following diabetic vitrectomy is not necessarily correlated with anatomic success. At the end stage of PDR, the detached retina loses the supply of choroidal nutrition, resulting in chronic microvascular deficiency of the retina (15). In addition, silicone oil filling is often needed for complex TRD, and because silicone oil has no substance exchange or metabolism, the likelihood of complications including high intraocular pressure, corneal decompensation, banded keratopathy, anterior uveitis, cataracts, optic neuropathy, and aseptic endophthalmitis, which all have an impact on visual function, increases over time (16–19). The prognostic factors included in our model are consistent with previous studies (20–22). Because of a long symptom duration and poor preoperative BCVA measurement, the diagnosis and treatment of PDR are not timely, resulting in irreversible retinal lesions. Moreover, hypertension can lead to thinning and distortion of the retinal artery, microangioma, retinal hemorrhage, retinal exudation, and optic disc edema; therefore, DR development as well as postoperative VH are both at danger because of it (23).

In addition to the four independent risk factors identified in this study, other factors have also been thought to affect visual acuity after diabetic vitrectomy. The progression of PDR is thought to be considerably influenced by the age at diabetes onset. Younger patients usually present with more severe anatomical features and have a higher rate of NVG and postoperative recurrent detachment (24). However, no previous study has reported that age affects postoperative vision recovery (6, 7, 24). Nevertheless, low vision in both eyes following diabetic vitrectomy is related to older age (8). In our study, advanced age only showed statistical significance in the univariate analysis. Therefore, although younger patients with PDR require more complex surgical procedures, their visual acuity can still be well recovered with proper intraoperative and postoperative management, whereas older patients tend to have worse functional outcomes.

CKD is also a concern for researchers; for instance, kidney failure was regarded as the most frequent cause of death in patients who underwent diabetic vitrectomy (25, 26). Complications such as anemia, hypoalbuminemia, and coagulation abnormality caused by CKD are also hazard elements exacerbating retinopathy (27). Furthermore, the same pathophysiological mechanism may link DR, CKD, and renal function impairment; particularly, low eGFR, contributes to the progression of DR (27–29). However, other studies have suggested that their relationship is not always consistent (30, 31). Regarding visual outcome, previous studies have not obtained positive results (32, 33). Moreover, our study showed that the groups did not significantly differ in baseline BCVA measurements or BCVA change; however, lower eGFR predicted a worse visual outcome. Regarding other factors, PDR patients with severe renal failure are more likely to undergo bilateral vitrectomy (34). Additionally, patients who have an eGFR below 60 ml/min/1.73 m^2^ are more likely to develop VH and NVG six months postoperatively (35). Our study also revealed that postoperative NVG was more common in patients with renal insufficiency; nonetheless, no significant difference was found in re-vitrectomy. In addition, many oculists worry that anticoagulant agents for hemodialysis would increase the possibility of postoperative bleeding (36). In our study, ten cases required dialysis, and only two of them developed postoperative VH, which was not statistically significant as a result of the limited sample size. Although patients with PDR with renal insufficiency are more likely to have worse visual outcomes and more complications, this outcome should not be regarded as a contraindication to PPV. Planned microinvasive PPV can help patients with PDR with renal insufficiency achieve the same visual improvement as patients with normal renal function.

Although previous studies have explored the prognostic factors of long-term visual acuity after diabetic vitrectomy (6–9, 11), few studies have established risk prediction models. Furthermore, only preoperative characteristics were included in this study, and the AUC was slightly better than the outcomes of logistic regression analysis in a recently published article (20), which included intraoperative data, such as silicone oil tamponade. Our model was further validated by adding an external validation cohort and expressed as nomogram; therefore, it may be more valuable in clinical application for doctors and patients to predict long-term visual outcomes before operation.

Postoperative long-term visual acuity is the most concerning issue for patients with PDR. In this study, a BCVA measurement of 0.3 was selected as the cutoff value according to the WHO 2015 standard. In clinical practice, we also found a BCVA measurement of 0.3 to be the critical value of subjective feeling for patients with PDR. Additionally, in this study, the surgical procedures were performed by one surgeon, eliminating the impact of skill level and treatment habits on the prognosis.

Nevertheless, this study had some limitations. First, confounding variables and bias are present owing to its retrospective nature. Second, we could not obtain comprehensive preoperative and follow-up data. Finally, this study did not consider the difference between 23-gauge and 25-gauge vitrectomy systems because previous studies have proved that the differences of these systems in terms of visual prognosis are negligible (37). Future multicenter research is expected to verify and optimize the model. Moreover, we will expand the sample size and establish predictive models using different outcomes. We also plan to include preoperative imaging data with the help of artificial intelligence.

In conclusion, TRD, symptom duration, preoperative BCVA measurement, and hypertension are independent risk factors of long-term low vision after diabetic vitrectomy. The risk prediction model based on these four risk factors exhibited good predictive value. This prognostic nomogram could help clinicians make better surgical decisions, assist patients with PDR in adjusting their psychological expectations, and foster better patient-doctor communication.

## Data availability statement

The raw data supporting the conclusions of this article will be made available by the authors, without undue reservation.

## Ethics statement

The studies involving human participants were reviewed and approved by Ethics Committee of Tianjin Medical University Eye Hospital. The patients/participants provided their written informed consent to participate in this study. Written informed consent was obtained from the individual(s) for the publication of any potentially identifiable images or data included in this article.

## Author contributions

BH, HG, ZW, and XL designed the study. HG and ZW wrote the manuscript. Data collection was performed by HG, ZW, ZN, XZ, KW, ND, SB, and WL. HG and ZW completed all statistical analysis. All authors contributed to the article and approved the submitted version.

## References

[1] Tsiknakis N Theodoropoulos D Manikis G Ktistakis E Boutsora O Berto A. Deep learning for diabetic retinopathy detection and classification based on fundus images: A review. Comput Biol Med (2021) 135:104599. doi: 10.1016/j.compbiomed.2021.104599 34247130

[2] Teo ZL Tham YC Yu M Chee ML Rim TH Cheung N. Global prevalence of diabetic retinopathy and projection of burden through 2045: systematic review and meta-analysis. Ophthalmology (2021) 128:1580–91. doi: 10.1016/j.ophtha.2021.04.027 33940045

[3] Flaxel CJ Adelman RA Bailey ST Fawzi A Lim JI Vemulakonda GA. Diabetic retinopathy preferred practice pattern®. Ophthalmol (2020) 127:P66–p145. doi: 10.1016/j.ophtha.2019.09.025 31757498

[4] Chaudhary S Zaveri J Becker N. Proliferative diabetic retinopathy (PDR). Dis Mon (2021) 67:101140. doi: 10.1016/j.disamonth.2021.101140 33546872

[5] Nakamura J Kamiya H Haneda M Inagaki N Tanizawa Y Araki E. Causes of death in Japanese patients with diabetes based on the results of a survey of 45,708 cases during 2001-2010: report of Committee on Causes of Death in Diabetes Mellitus. Diabetol Int (2017) 8:117–36. doi: 10.1007/s13340-017-0313-3 PMC6224959 30603315

[6] Nishi K Nishitsuka K Yamamoto T Yamashita H. Factors correlated with visual outcomes at two and four years after vitreous surgery for proliferative diabetic retinopathy. PLoS One (2021) 16:e0244281. doi: 10.1371/journal.pone.0244281 33444332 PMC7808600

[7] Ostri C Lux A Lund-Andersen H la Cour M. Long-term results, prognostic factors and cataract surgery after diabetic vitrectomy: a 10-year follow-up study. Acta Ophthalmol (2014) 92:571–6. doi: 10.1111/aos.12325 24373516

[8] Schreur V Brouwers J Van Huet RAC Smeets S Phan M Hoyng CB. Long-term outcomes of vitrectomy for proliferative diabetic retinopathy. Acta Ophthalmol (2021) 99:83–9. doi: 10.1111/aos.14482 PMC7891313 32643273

[9] Mason JO 3rd Colagross CT Haleman T Fuller JJ White MF Feist RM. Visual outcome and risk factors for light perception and no light perception vision after vitrectomy for diabetic retinopathy. Am J Ophthalmol (2005) 140:231–5. doi: 10.1016/j.ajo.2005.02.052 15992755

[10] Levey AS Stevens LA Schmid CH Zhang YL Castro AF 3rd Feldman 3HI. A new equation to estimate glomerular filtration rate. Ann Intern Med (2009) 150:604–12. doi: 10.7326/0003-4819-150-9-200905050-00006 PMC2763564 19414839

[11] Gupta B Wong R Sivaprasad S Williamson TH. Surgical and visual outcome following 20-gauge vitrectomy in proliferative diabetic retinopathy over a 10-year period, evidence for change in practice. Eye (Lond) (2012) 26:576–82. doi: 10.1038/eye.2011.348 PMC3325568 22241020

[12] Levey AS Eckardt KU Tsukamoto Y Levin A Coresh J Rossert J. Definition and classification of chronic kidney disease: a position statement from Kidney Disease: Improving Global Outcomes (KDIGO). Kidney Int (2005) 67:2089–100. doi: 10.1111/j.1523-1755.2005.00365.x 15882252

[13] Mikhail M Ali-Ridha A Chorfi S Kapusta MA. Long-term outcomes of sutureless 25-G+ pars-plana vitrectomy for the management of diabetic tractional retinal detachment. Graefes Arch Clin Exp Ophthalmol (2017) 255:255–61. doi: 10.1007/s00417-016-3442-7 27480177

[14] Meleth AD Carvounis PE. Outcomes of vitrectomy for tractional retinal detachment in diabetic retinopathy. Int Ophthalmol Clin (2014) 54:127–39. doi: 10.1097/IIO.0000000000000021 24613889

[15] Bornfeld N Bechrakis NE. Ophthalmological oncology. Klin Monbl Augenheilkd (2011) 228:585. doi: 10.1055/s-0031-1281585 21739399

[16] Miller JB Papakostas TD Vavvas DG. Complications of emulsified silicone oil after retinal detachment repair. Semin Ophthalmol (2014) 29:312–8. doi: 10.3109/08820538.2014.962181 25325856

[17] Morphis G Irigoyen C Eleuteri A Stappler T Pearce I Heimann H. Retrospective review of 50 eyes with long-term silicone oil tamponade for more than 12 months. Graefes Arch Clin Exp Ophthalmol (2012) 250:645–52. doi: 10.1007/s00417-011-1873-8 22138760

[18] Toklu Y Cakmak HB Ergun SB Yorgun MA Simsek S. Time course of silicone oil emulsification. Retina (2012) 32:2039–44. doi: 10.1097/IAE.0b013e3182561f98 22653542

[19] Do DV Gichuhi S Vedula SS Hawkins BS. Surgery for postvitrectomy cataract. Cochrane Database Syst Rev (2018) 1:Cd006366. doi: 10.1002/14651858.CD006366.pub4 29364503 PMC6491312

[20] Lee SS Chang DJ Kwon JW Min JW Jo K Yoo YS. Prediction of visual outcomes after diabetic vitrectomy using clinical factors from common data warehouse. Transl Vis Sci Technol (2022) 11:25. doi: 10.1167/tvst.11.8.25 PMC9428357 36006638

[21] Gupta B Sivaprasad S Wong R Laidlaw A Jackson TL McHugh D. Visual and anatomical outcomes following vitrectomy for complications of diabetic retinopathy: the DRIVE UK study. Eye (Lond) (2012) 26:510–6. doi: 10.1038/eye.2011.321 PMC3325558 22222268

[22] Yorston D Wickham L Benson S Bunce C Sheard R Charteris D. Predictive clinical features and outcomes of vitrectomy for proliferative diabetic retinopathy. Br J Ophthalmol (2008) 92:365–8. doi: 10.1136/bjo.2007.124495 18303158

[23] Lingam G Wong TY. Systemic medical management of diabetic retinopathy. Middle East Afr J Ophthalmol (2013) 20:301–8. doi: 10.4103/0974-9233.120010 PMC3841947 24339679

[24] Huang CH Hsieh YT Yang CM. Vitrectomy for complications of proliferative diabetic retinopathy in young adults: clinical features and surgical outcomes. Graefes Arch Clin Exp Ophthalmol (2017) 255:863–71. doi: 10.1007/s00417-016-3579-4 28063082

[25] Banerjee PJ Moya R Bunce C Charteris DG Yorston D Wickham L. Long-term survival rates of patients undergoing vitrectomy for proliferative diabetic retinopathy. Ophthalmic Epidemiol (2016) 23:94–8. doi: 10.3109/09286586.2015.1089578 26954846

[26] Liu E Estevez J Kaidonis G Hassall M Phillips R Raymond G. Long-term survival rates of patients undergoing vitrectomy for diabetic retinopathy in an Australian population: a population-based audit. Clin Exp Ophthalmol (2019) 47:598–604. doi: 10.1111/ceo.13466 30663192

[27] Zhang J Wang Y Li L Zhang R Guo R Li H. Diabetic retinopathy may predict the renal outcomes of patients with diabetic nephropathy. Ren Fail (2018) 40:243–51. doi: 10.1080/0886022X.2018.1456453 PMC6014304 29633887

[28] Wang J Xin X Luo W Wang R Wang X Si S. Anemia and diabetic kidney disease had joint effect on diabetic retinopathy among patients with type 2 diabetes. Invest Ophthalmol Vis Sci (2020) 61:25. doi: 10.1167/iovs.61.14.25 PMC7757636 33351059

[29] Cho A Park HC Lee YK Shin YJ Bae SH Kim H. Progression of diabetic retinopathy and declining renal function in patients with type 2 diabetes. J Diabetes Res (2020) 2020:8784139. doi: 10.1155/2020/8784139 32802891 PMC7403926

[30] Grunwald JE Pistilli M Ying GS Daniel E Maguire M Xie D. Association between progression of retinopathy and concurrent progression of kidney disease: findings from the chronic renal insufficiency cohort (CRIC) study. JAMA Ophthalmol (2019) 137:767–74. doi: 10.1001/jamaophthalmol.2019.1052 PMC6512259 31070679

[31] Zhao L Ren H Zhang J Cao Y Wang Y Meng D. Diabetic retinopathy, classified using the lesion-aware deep learning system, predicts diabetic end-stage renal disease in chinese patients. Endocr Pract (2020) 26:429–43. doi: 10.4158/EP-2019-0512 31968187

[32] Larrañaga-Fragoso P Laviers H McKechnie C Zambarakji H. Surgical outcomes of vitrectomy surgery for proliferative diabetic retinopathy in patients with abnormal renal function. Graefes Arch Clin Exp Ophthalmol (2020) 258:63–70. doi: 10.1007/s00417-019-04532-7 31758258

[33] Rice JC Steffen J. Outcomes of vitrectomy for advanced diabetic retinopathy at Groote Schuur Hospital, Cape Town, South Africa. S Afr Med J (2015) 105:496–9. doi: 10.7196/SAMJ.9203 26716170

[34] Song YS Nagaoka T Omae T Yokota H Takahashi A Yoshida A. Systemic risk factors in bilateral proliferative diabetic retinopathy requiring vitrectomy. Retina (2016) 36:1309–13. doi: 10.1097/IAE.0000000000000886 26630317

[35] Kameda Y Saeki T Hanai K Suzuki Y Uchigata Y Babazono T. Is chronic kidney disease affecting the postoperative complications of vitrectomy for proliferative diabetic retinopathy? J Clin Med (2021) 10:5309. doi: 10.3390/jcm10225309 34830589 PMC8621452

[36] Janssen MJ van der Meulen J. The bleeding risk in chronic haemodialysis: preventive strategies in high-risk patients. Neth J Med (1996) 48:198–207. doi: 10.1016/0300-2977(96)00005-8 8710039

[37] Ding Y Yao B Hang H Ye H. Multiple factors in the prediction of risk of recurrent vitreous haemorrhage after sutureless vitrectomy for non-clearing vitreous haemorrhage in patients with diabetic retinopathy. BMC Ophthalmol (2020) 20:292. doi: 10.1186/s12886-020-01532-8 32677996 PMC7367221

